# Mechanism of Ghrelin-Induced Gastric Contractions in *Suncus murinus* (House Musk Shrew): Involvement of Intrinsic Primary Afferent Neurons

**DOI:** 10.1371/journal.pone.0060365

**Published:** 2013-04-02

**Authors:** Anupom Mondal, Sayaka Aizawa, Ichiro Sakata, Chayon Goswami, Sen-ichi Oda, Takafumi Sakai

**Affiliations:** 1 Area of Regulatory Biology, Graduate School of Science and Engineering, Saitama University, Saitama, Japan; 2 Laboratory of Animal Management and Resources, Department of Zoology, Okayama University of Science, Okayama, Japan; Goethe University Frankfurt, Germany

## Abstract

Here, we have reported that motilin can induce contractions in a dose-dependent manner in isolated *Suncus murinus* (house musk shrew) stomach. We have also shown that after pretreatment with a low dose of motilin (10^−10^ M), ghrelin also induces gastric contractions at levels of 10^−10^ M to 10^−7^ M. However, the neural mechanism of ghrelin action in the stomach has not been fully revealed. In the present study, we studied the mechanism of ghrelin-induced contraction *in vitro* using a pharmacological method. The responses to ghrelin in the stomach were almost completely abolished by hexamethonium and were significantly suppressed by the administration of phentolamine, prazosin, ondansetron, and naloxone. Additionally, *N*-nitro-l-arginine methylester significantly potentiated the contractions. Importantly, the mucosa is essential for ghrelin-induced, but not motilin-induced, gastric contractions. To evaluate the involvement of intrinsic primary afferent neurons (IPANs), which are multiaxonal neurons that pass signals from the mucosa to the myenteric plexus, we examined the effect of the IPAN-related pathway on ghrelin-induced contractions and found that pretreatment with adenosine and tachykinergic receptor 3 antagonists (SR142801) significantly eliminated the contractions and GR113808 (5-**hydroxytryptamine** receptor 4 antagonist) almost completely eliminated it. The results indicate that ghrelin stimulates and modulates suncus gastric contractions through cholinergic, adrenergic, serotonergic, opioidergic neurons and nitric oxide synthases in the myenteric plexus. The mucosa is also important for ghrelin-induced gastric contractions, and IPANs may be the important interneurons that pass the signal from the mucosa to the myenteric plexus.

## Introduction

Ghrelin is an orexigenic peptide isolated from the stomach as an endogenous ligand for growth hormone secretagogue receptors (GHS-Rs) [1]. Ghrelin is mainly involved in the stimulation of growth hormone secretion [1] and food intake [2] and forms a peptide family with motilin owing to their similarities in not only peptides but also receptors [3]. Moreover, endogenous ghrelin is generally considered to regulate gastric contractions in the interdigestive state [4]; administration of ghrelin stimulates gastric contractions in rats [5], mice [6], and humans [7] and may be an alternative to motilin for gastrointestinal (GI) motility in motilin-lacking rodents [3]. However, the mechanism underlying the role played by ghrelin in the regulation of fasted motor activities in the GI tract is not fully understood.

Functional analyses of ghrelin-induced GI motor-stimulating actions both *in vivo* and *in vitro* have suggested 2 main mechanisms for these responses. Ghrelin stimulates fasted intestinal motor activity in rats through ghrelin receptors on vagal afferent nerves [8]. Moreover, the gastric motor-stimulating action of ghrelin in rats shows vagovagal sensitivity [5], [9]. The expression of ghrelin receptors in the nodose ganglion [10], [11] and the capability of ghrelin to modify the discharges of afferent vagal neurons [12] also support the essential role of a vagovagal reflex pathway in ghrelin-induced responses. In addition to this reflex pathway is a mechanism via direct activation of the enteric nervous system in ghrelin-stimulated contraction. In rats and mice, the gastroprokinetic activity of ghrelin is observed *in vitro* as an increase in neuronally mediated contractions evoked by electrical field stimulation (EFS) [9], [13], [14], [15], [16], [17], and a ghrelin-induced fasted motor pattern has also been observed in vagotomized rats [8]. Together, these results suggest that at least one of the target sites of ghrelin in rodents is the enteric nervous system. However, the phenotypes of ghrelin-sensitive enteric nerves have not been clearly described to date.

One explanation for the gap in information is that the effects of ghrelin activity have thus far been investigated using EFS systems in the case of smooth muscle preparations [9], [13], [14], [15], [16], [17]. Therefore, the presence of a complete neural package in the stomach has not been studied. Moreover, the actions of ghrelin are species dependent, similar to those of the ghrelin-related peptide motilin. For example, ghrelin does not stimulate canine and rabbit GI motility [7], [18] but induces gastric contractions in rats, mice, and humans, and although motilin stimulates GI motility in rabbits [19], dogs [20], and humans [21], it has no effect in mice and rats. To address these dissimilarities, we used *Suncus murinus* (house musk shrew) in an organ bath study. *S. murinus* belongs to the order Insectivora, family Soricidae, and this order of animals is considered one of the key groups for understanding the origin of mammals [22], [23]. We have already identified the complementary DNA sequences of suncus motilin and ghrelin in *S. murinus* using polymerase chain reaction cloning [24], [25]. We have also identified GHS-R and G protein-coupled receptor 38 genes in *S. murinus* [26]. Moreover, we have studied the contractile properties of the stomach in conscious, free-moving *S. murinus* as well as in organ-bath experiments and found that *S. murinus* has GI motility that is almost identical to that in humans and dogs [24], [27]. We have also published the mechanism of motilin-induced gastric contractions in the *S. murinus* stomach [28].

Recently, we demonstrated that ghrelin can induce gastric contractions after pretreatment with a low dose of motilin, and this coordination of motilin and ghrelin may be necessary for the initiation of phase III contractions [29]. However, the mechanism and neural pathway of that synergistic effect in the enteric nervous system is unknown. To clarify this point, we investigated the mechanism of ghrelin-induced contractions *in vitro* using the whole stomach of *S. murinus*.

## Materials and Methods

### Animals

The experiments were conducted using female suncuses (weight, 45–75 g) older than 5 weeks, from an outbred KAT strain established from a wild population in Kathmandu, Nepal [30]. Animals were housed individually in plastic cages equipped with empty cans for nest boxes. The animals were given food (trout pellets; Nippon Formula Feed Manufacturing Co. Ltd., Yokohama, Japan) and water *ad libitum*. The animal room was maintained at 21°C to 24°C and the light-dark cycle was controlled to change every 12 h (lights on from 0800 to 2000 h). All procedures were approved by and performed in accordance with the guidelines of the Saitama University Committee on Animal Research. All efforts were made to minimize animal suffering and reduce the number of animals used in the experiment.

### Preparation of Suncus Isolated Stomach

After being deeply anesthetized with diethyl ether, the animals were killed via decapitation, and their stomachs were immediately placed into freshly prepared Krebs solution (composition in mM: NaCl, 118; KCl, 4.75; CaCl_2_, 2.5; MgSO_4_, 1.2; NaH_2_PO_4_, 1.8; NaHCO_3_, 25; and glucose, 11.5; pH 7.2) after laparotomy. The mesentery attachments and fatty tissues were removed, and the inside of each stomach was washed with Krebs solution through a small incision in the gastric fundus. The stomachs were then mounted in 10-mL water-jacketed organ baths and initially loaded with weight totaling approximately 1.0 g. The temperature of the Krebs solution was maintained at 37°C ±0.5°C, and the solution was aerated continuously with a mixture of 95% O_2_ and 5% CO_2_.

### Gastric Contractility Study

The contractile activities of the stomach in response to ghrelin treatment were monitored using an isometric force transducer (UM-203; Iwashiya Kishimoto Medical Instruments, Kyoto, Japan) and software (PicoLog for Windows, Pico Technology Ltd., St. Neots, UK). To normalize the contractions in this experiment, we added acetylcholine (ACh; 10^−5^ M) to the organ bath twice before the cumulative administration of ghrelin (after pretreatment with 10^−10^ M motilin) in the absence and presence of an antagonist. At the end of the experiment, ACh (10^−5^ M) was added once again to the organ bath, and the percentage of maximal contractions was calculated by averaging the tonic response induced by these 3 administrations. Note that in each case, the ACh administration evoked almost the same tonic gastric contractions. Then the effects of acyl ghrelin in the absence or presence of antagonists were expressed as a percentage of the control contractions. Concentration-response curves were obtained through cumulative addition of acyl ghrelin with or without antagonists or an inhibitor at appropriate intervals to the organ bath.

### Damaging the Mucosa

The mucosa was damaged mechanically. In brief, the ghrelin-induced (with low-dose motilin pretreatment) and motilin-induced gastric contractions were recorded as a control, the stomach was removed from the organ bath, and the mucosa was damaged through gentle pressing with cover-glass forceps. These damaged stomachs were loaded into the organ bath and washed several times with Krebs buffer. The stomachs were again mounted in 10-mL water-jacketed organ baths, and the contractile properties were measured using ghrelin and motilin.

### Drugs Used

Acetylcholine chloride (Sigma, USA) was dissolved in distilled water, and synthetic suncus motilin (Bex, Tokyo, Japan) was dissolved in 0.1% bovine serum albumin/phosphate-buffered saline. In antagonist or inhibitor experiments, the stomachs were equilibrated before the application of acyl ghrelin (pretreated with a low dose of motilin) with the following antagonists: hexamethonium bromide (10^−4^ M; Wako, Osaka, Japan) [31], prazosin hydrochloride (10^−6^ M; Wako) [32], timolol maleate (10^−6^ M; Wako) [33], naloxone (10^−6^ M; Wako) [34], FK888 (10^−6^ M; Tocris Bioscience, Ellisville, USA) [35], ondansetron (10^−5^ M; Hikari Pharmaceutical, Imado, Japan) [36], and phentolamine mesylate (10^−5^ M; MP Biomedicals, France) [32] for 30 min; yohimbine hydrochloride (10^−6^ M; Tocris Bioscience) [33] for 25 min; ritanserin (10^−7^ M; Tocris Bioscience) [37] for 1 h, or *N*-nitro-l-arginine methylester (L-NAME; 10^−4^ M; Sigma) [38], adenosine (10^−8.5^ M; Sigma) [39], and GR113808 (10^−7^ M; Tocris Bioscience) [40] for 15 min; and SR142801 (10^−7^ M; Axon Medchem, Groningen, The Netherlands) [41] for 20 min. Concentrations of drugs were expressed as final molar concentrations in the bath solution. Ritanserin and FK888 were dissolved in ethanol, and the other compounds were dissolved in distilled water before use. All reagents were prepared for each experiment according to manufacturer instructions.

### Statistical Analysis

The results of experiments have been expressed as mean ± standard error of the mean values of more than 4 separate experiments using whole stomachs. One-way analysis of variance followed by the Student’s *t*-test was used for the statistical analysis of data. P<0.05 and P<0.01 were considered significant.

## Results

### Cholinergic Pathway

**Figure 1:**
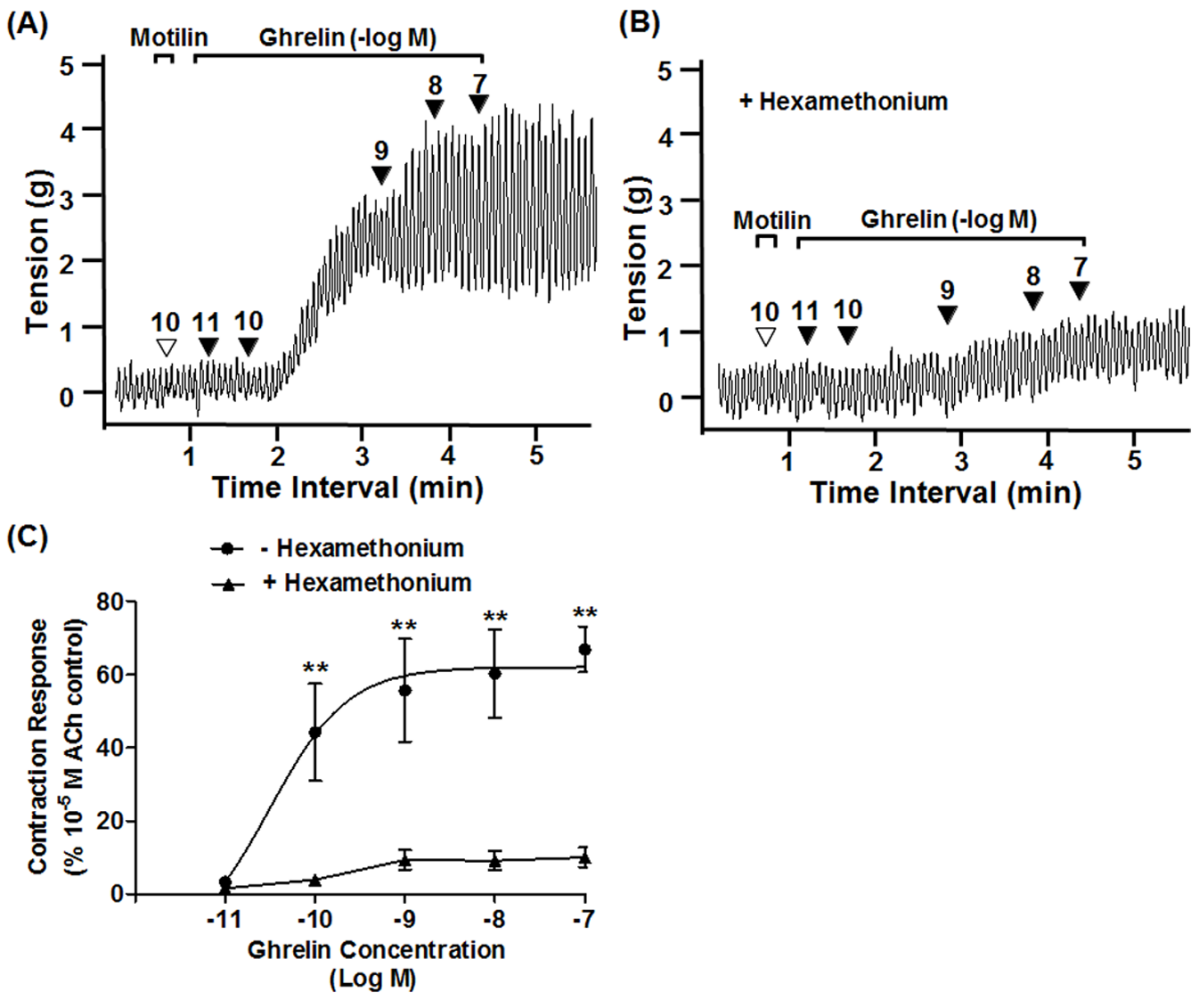
Cholinergic pathway of ghrelin-induced suncus gastric contractions. (A) After pretreatment with motilin (10^−10^ M), ghrelin stimulated gastric contractions from 10^−10^ M. (B) Trace showing that ghrelin-induced gastric contractions was considerably attenuated by pretreatment with hexamethonium (10^−4^ M). (C) Concentration-response curve showing the almost complete inhibitory effect of hexamethonium on ghrelin-induced gastric contractions. ▾, timing of the administration of reagents; the number is the concentration of ghrelin (-Log[ghrelin]M). Each value is the mean ± standard error of the mean (SEM; n = 6). •: Control; **▴**: antagonist treatment; ^**^P<0.01. ACh, acetylcholine.

We have previously shown that ghrelin induces gastric contractions after pretreatment with a low dose of motilin (10^−10^ M) [42]. We have also reported that atropine, a muscarinic receptor antagonist, completely abolishes the response to ghrelin in the stomach and suppresses spontaneous contractile activity [42]. Similarly, hexamethonium, a nicotinic receptor antagonist, almost completely eliminates contraction induced by ghrelin (10^−10^–10^−9^ M; Figure 1A–C) and hexamethonium reduced the effect of 10^−7^ M dose of ghrelin by 85.1±3.2%.

### Adrenergic Pathway

**Figure 2:**
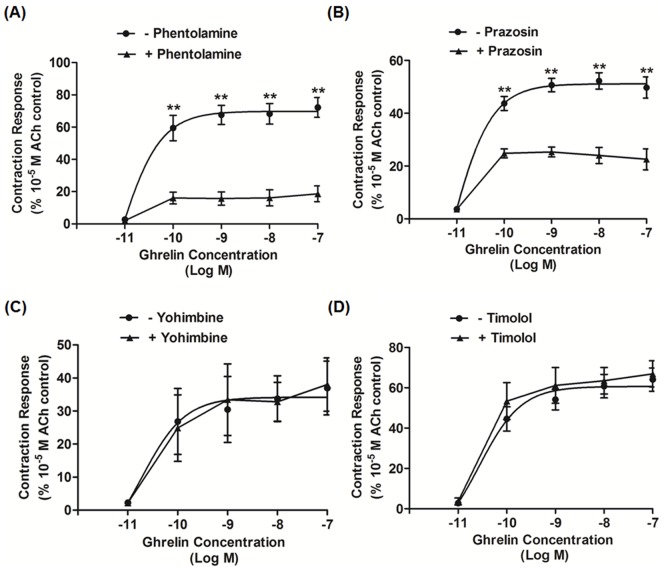
Effects of pretreatment with adrenergic receptor antagonists on ghrelin-induced suncus gastric contractions. (A) Phentolamine (α adrenergic receptor antagonist; 10^−5^ M) significantly decreased contractions. (B) Prazosin (α1 adrenergic receptor antagonist; 10^−6^ M) also significantly suppressed the control contractions. (C) Yohimbine (α2 adrenergic receptor antagonist; 10^−6^ M) had no significant effect. (D) Timolol (β adrenergic receptor antagonist; 10^−6^ M) had no effect. Each value is the mean ± SEM (n = 6). •: Control; **▴**: antagonist treatment;^ **^P<0.01. ACh, acetylcholine.

We also examined the involvement of adrenergic neurons. Phentolamine, an α receptor antagonist, markedly inhibited ghrelin-induced contraction (Figure 2A) and decreased spontaneous contraction (data not shown). Ghrelin-induced tonic contraction was also significantly suppressed by pretreatment with prazosin, an α_1_ receptor antagonist (Figure 2B). Phentolamine and prazosin reduced the stimulatory effect of 10^−7^ M dose of ghrelin by 74.1±6.3% and 55.6±6.5%, respectively. Conversely, yohimbine, an α_2_ receptor antagonist, and timolol, a β receptor antagonist, did not affect ghrelin stimulatory contraction (Figure 2C, D).

### Serotonergic Pathway

**Figure 3:**
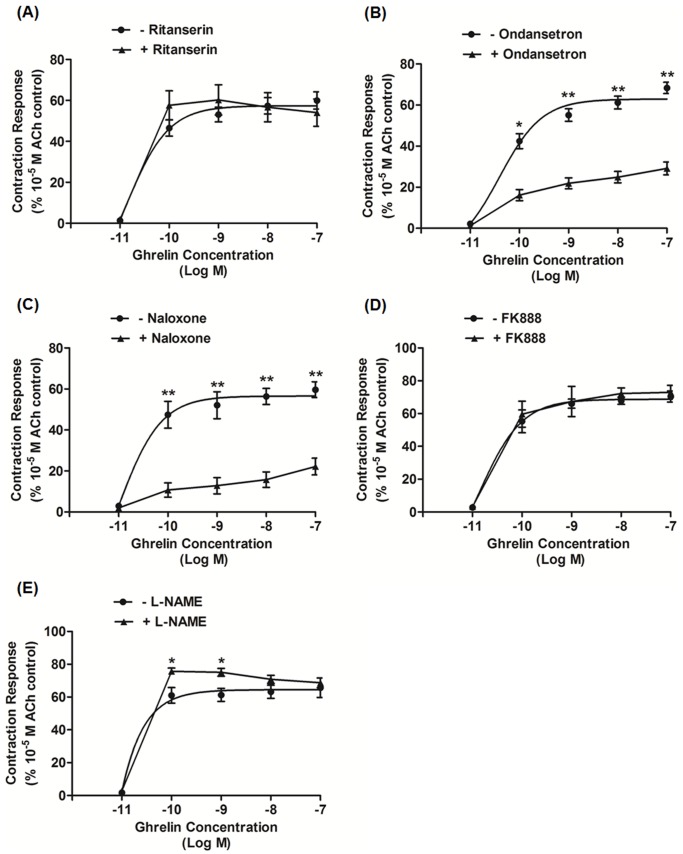
Ghrelin-induced concentration-response curves and effects of pretreatment with ritanserin (5-hydroxytryptamine [HT] 2 receptor antagonist), ondansetron (5HT_3_ receptor antagonist), naloxone (µ opioid receptor antagonist), FK888 (neurokinin [NK] 1 receptor antagonist), and *N*-nitro-l-arginine methylester (L-NAME; nitrous oxide synthase inhibitor). (A) Ritanserin (10^−7^ M) showed no antagonistic effect on ghrelin-induced contractions. (B) Ondansetron treatment (10^−5^ M) significantly reduced ghrelin-induced contractions. (C) Naloxone (10^−6^ M) significantly inhibited ghrelin-induced contractions. (D) FK888 had no significant effect on ghrelin-induced contractions. (E) L-NAME significantly potentiated the contractions at doses of 10^−10^ and 10^−9^ M ghrelin. Each value is the mean ± SEM value (n = 6). •: Control; ▴: antagonists or inhibitor treatment; ^*^P<0.05, ^**^P<0.01. ACh, acetylcholine.

In the present study, ritanserin, a 5-hydroxytryptamine **(**HT) 2 receptor antagonist, did not decrease ghrelin-induced contractions at any concentration (Figure 3A) but markedly suppressed spontaneous contractile activity (data not shown). In contrast to ritanserin, ondansetron, a 5-HT_3_ receptor antagonist, significantly suppressed the contractions induced by ghrelin (Figure 3B) and the rate (%) of reduction by the ondansetron on the stimulatory effects of 10^−7^ M dose of ghrelin was 58.1±3.2%.

### Other Receptor Antagonists and Nitric Oxide (NO) Synthase Inhibitor

The effects of other receptor antagonists and NO synthase inhibitors were also investigated for further characterization of the ghrelin response. Naloxone, an opiate receptor antagonist, potentially suppressed the contractions induced by ghrelin (Figure 3C) but did not affect spontaneously occurring phasic contractions (data not shown) and the inhibitory rate (%) of the naloxone on the effect of 10^−7^ M ghrelin was 62.9±6.1%. Neither spontaneously occurring contractions nor motilin-induced contractions were decreased by FK888, a neurokinin (NK) 1 receptor antagonist (Figure 3D). *N*-nitro-l-arginine methylester, an inhibitor of NO synthase, potentiated the contractions induced by 10^−10^ and 10^−9^ M ghrelin but did not significantly change contractions induced by other ghrelin concentrations (Figure 3E).

### Importance of Mucosa in Ghrelin-induced Contraction

**Figure 4:**
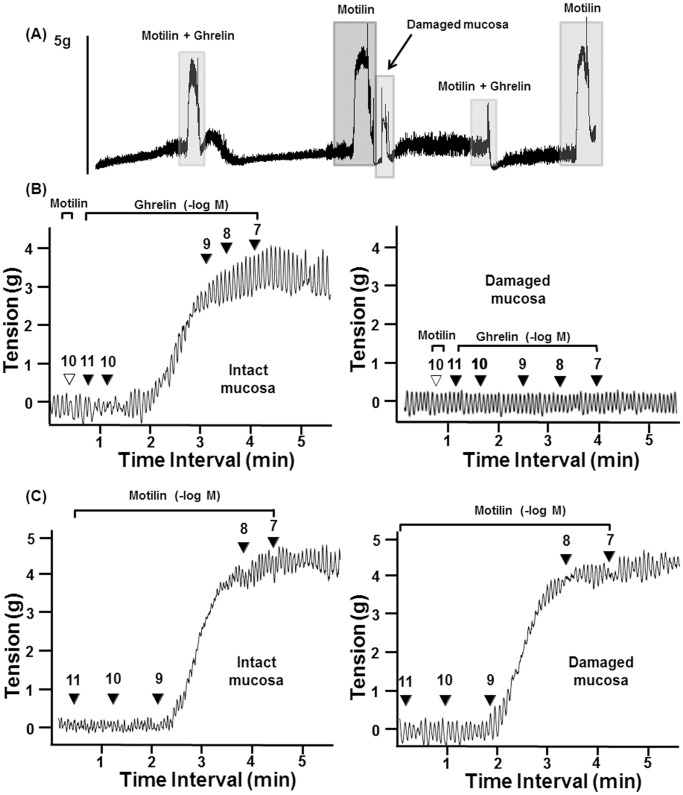
Involvement of the mucosa in ghrelin-induced contractions. (A) Chart showing the increasing pattern of ghrelin-induced (after pretreatment with a low dose of motilin) and motilin-induced gastric contractions before and after damage to the mucosa. (B) Magnification of part A showing that ghrelin can stimulate gastric contractions with the mucosa intact, but gastric contraction was absent after damage to the mucosal layers. (C) Motilin evoked the same gastric contraction even after mucosal damage. ▾, timing of the administration of reagents; the number is the concentration of ghrelin (-Log[ghrelin]M; n = 4). ACh, acetylcholine.

Because ghrelin-producing cells have been observed in the gastric mucosa of *S. murinus* [25], we predicted that the mucosa plays an important role in the ghrelin stimulatory pathway. Figure 4A depicts the synergistic effect of motilin and ghrelin with and without damage to the gastric mucosa. Interestingly, after the breakdown in mucosa, ghrelin-induced gastric contraction was completely abolished (see Figure 4A, B), whereas motilin-induced contraction was preserved (see Figure 4A, C), suggesting that mucosa has a central role in triggering ghrelin-induced contractions.

### Effects of Adenosine, SR142801, and GR113808 on Ghrelin-induced Contraction

**Figure 5:**
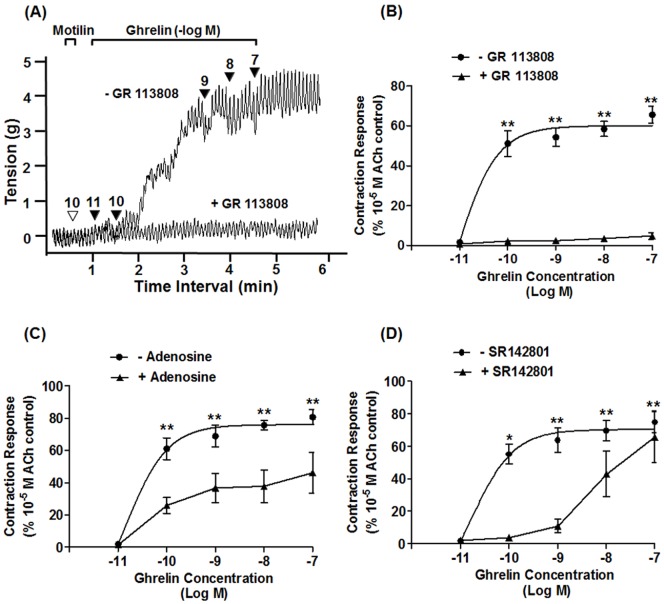
Effects of GR113808 (5HT_4_ receptor antagonist), adenosine (adenosine receptor agonist), and SR142801 (NK_3_ receptor antagonist) on ghrelin-induced contractions. (A) Traces of ghrelin-induced contractions with and without pretreatment with GR113808 (10^−7^ M). (B) Concentration-response curve showing that GR113808 completely abolished ghrelin-induced contractions. (C) Concentration-response curve showing that adenosine significantly attenuated ghrelin-induced contractions. (D) SR142801 almost completely inhibited ghrelin-induced gastric contraction at doses of 10^−10^ and 10^−9^ M ghrelin. ▾, timing of the administration of reagents; the number is the concentration of ghrelin (-Log[ghrelin]M). Each value is the mean ± SEM (n = 4). •: Control; ▴: antagonist treatment;^ *^P<0.05;^ **^P<0.01. ACh, acetylcholine.

Intrinsic primary afferent neurons (IPANs) reportedly play a pivotal role in controlling the motility in the GI tract through synaptic connections with other neurons in the myenteric, submucosal, and mucosal ganglia [43], [44]. Therefore, we predicted that IPANs play a significant role in the ghrelin regulatory pathway. IPAN is the primary transmitter of ACh and tachykinin [39], [44]. The 5HT_4_ receptor is also expressed on IPANs [45]. To confirm the involvement of these factors, we used pretreatment with GR113808 (5HT_4_ receptor antagonist), adenosine, an agonist of adenosine receptors (to inhibit the release of ACh from IPANs through A1 receptors), and SR142801 (NK_3_ receptor antagonist), and found that GR113808 completely eliminated ghrelin-induced contractions (Figure 5A, B). The concentration-response curve showed that adenosine pretreatment significantly abolished ghrelin-induced gastric contraction (Figure 5C). Pretreatment with SR142801 almost completely inhibited ghrelin-induced contractions at ghrelin doses of 10^−10^ and 10^−9^ M and significantly suppressed it at a concentration of 10^−8^ M ghrelin (Figure 5D). GR113808 and adenosine reduced the effects of 10^−7^ M dose of ghrelin by 92.6±2.5% and 39.9±18.7%, respectively, and SR142801 reduced the effects of 10^−9^ M ghrelin by 82.1±7.3%.

## Discussion

Since the discovery of ghrelin, the mechanisms of ghrelin activity with respect to GI motility have been increasingly investigated; however, clear insights into these mechanisms have been elusive thus far. In the present study, we used the stomach of *S. murinus*, a new model animal for the study of motilin/ghrelin, and revealed mechanisms of ghrelin-induced contractions with a pharmacological *in vitro* method. To investigate the response of the neural network to ghrelin in the enteric nervous system, we examined the effects of various receptor antagonists and a NO synthase inhibitor on ghrelin-induced contractions and characterized the pharmacological properties in the suncus stomach *in vitro*.

We have already reported the novel observation that ghrelin (10^−10^ to 10^−7^ M) can induce contraction in isolated *S. murinus* stomach in a dose-dependent manner when pretreated with a low concentration of motilin (10^−10^ M) [29]. Ghrelin-induced *S. murinus* gastric contractions have also been confirmed to operate in a vagus-independent manner [29]. In the present study, hexamethonium, a ganglion-blocking agent, almost completely suppressed the action of ghrelin and, as we have reported in a previous study, atropine also completely inhibits ghrelin-induced gastric contractions [29]. Several functional *in vivo* and *in vitro* studies have reported that the cholinergic system may be the dominant motor pathway in ghrelin-induced contractions [5], [17]. These results together indicate that myenteric preganglionic cholinergic neurons and postganglionic cholinergic neurons are equally important for ghrelin-induced gastric contractions. Moreover, given the inhibitory potency of hexamethonium (Table S1), presynaptic cholinergic activation plays a much more prominent role than that of motilin in ghrelin-induced gastric contraction [28].

The notable inhibitory effects of phentolamine and prazosin indicate that α receptors, specifically α_1_ receptors, but not α2 and β receptors, are also involved in ghrelin-induced contraction. Conversely, our previous study clearly showed that α2 receptors are important for motilin-induced gastric contraction [28]. Taken together, these results suggest that different neural pathways exist between ghrelin- and motilin-induced gastric contractions.

The significance of 5HT neurons has been observed in intestinal motility with vagus dependency [46], [47]. By contrast, we showed that 5HT3 but not 5HT2 neurons are involved in ghrelin-induced contractions with vagus independence. Similarly, for the first time, we observed that the opioidergic pathway has a vital role in ghrelin-stimulated contraction. Moreover, compared with motilin-induced gastric contraction in *S. murinus* [28], ghrelin-induced gastric contractions is strongly suppressed by ondansetron and naloxone pretreatment (see Table S1). Bassil *et al*. [16] have reported an EFS study in which ghrelin-induced contractions was evoked with multiple neurotransmitters. Ghrelin increases EFS-evoked contraction mediated by a combination of cholinergic and non-cholinergic excitatory activity in rats [15]. These results suggest that not only pre-ganglionic cholinergic neurons but also the involvement of 5HT3 and opioidergic neurons are likely of similar importance for ghrelin-induced contraction in *S. murinus* stomach.

Another significant observation is that motilin is essential for ghrelin-induced gastric contractions [42]. Ghrelin administration alone, despite the administration of high doses (10^−7^ M), failed to stimulate any gastric contractions; however, pretreatment with a low dose of motilin restored sensitivity to ghrelin, implying that motilin opens the gate of the ghrelin pathway. Two possibilities explain this gate-opening property: (1) motilin may synergistically stimulate the ghrelin pathway, or (2) motilin may produce inhibitory effects on some strong suppression mechanism. Considering the differing properties between the independent administration of ghrelin (1000 times higher doses) and motilin-pretreated ghrelin, which can induce gastric contractions (i.e., 10^−7^ M versus 10^−10^ M ghrelin), it is reasonable to predict that in restoring ghrelin activity, a low dose of motilin may affect the inhibitory neural network in the myenteric plexus in a flip-flop manner. However, we cannot draw any conclusions at this time, and therefore, this area remains an important one for further investigation.

Apart from this, the most interesting finding in this study is the significance of the mucosa for ghrelin-induced, but not motilin-induced, gastric contractions. We have reported that ghrelin-producing cells are found in suncus gastric mucosa [25] and that GHS-R messenger RNA expression is found in both the mucosa and the muscle layers of *S. murinus* stomach [26]. Therefore, some specialized neurons may responsible for the transfer of signals from the mucosa to the myenteric plexus in ghrelin-induced gastric contraction. Mucosal stimulation is thought to activate IPANs in both the submucosal [48], [49], [50], [51] and the myenteric plexuses [39], [52], [53], and this distinctive shape is known as Dogiel type II morphology [44]. Therefore, we assumed that the IPANs may act as interneurons in ghrelin stimulatory action. The primary transmit component of IPANs are ACh and tachykinergic neurons [44]. Moreover, adenosine activates adenosine receptors, and most afterhyperpolarizing/Dogiel type II neurons (about 85%) are hyperpolarized by adenosine treatment–largely through A1 receptors [54] via inhibition of ACh release in the myenteric plexus [55]–and many studies have shown that the cell bodies of myenteric IPANs bear NK3 receptors [56], [57], [58], [59]. In the present study, we found that pretreatment with adenosine significantly suppressed ghrelin-induced gastric contractions and that an NK3 receptor antagonist (SR142801) almost completely abolished the contraction at doses of 10^−10^ and 10^−9^ M. Conversely, we also observed that both adenosine and SR142801 had no effect on motilin-induced gastric contractions (Figures S1A, B). Although GR113808 (5HT4 receptor antagonist) treatment completely inhibited ghrelin-induced contraction, motilin-induced contraction was partially suppressed by GR113808 treatment (Figure S2). 5HT_4_ receptors are reportedly present in IPANs and in some other neuron types in the myenteric plexus [45]. Recently, Takahashi *et al.* [60] observed that 5HT_4_ receptors are important in regulating migrating motor complexes through IPANs. Taken together, these data indicate that a different stimulatory pathway exists between the motilin and the ghrelin effects and that IPANs may be implicated in the effects of ghrelin on GI motility.

IPANs with cell bodies in myenteric ganglia have been shown to transmit via slow excitatory postsynaptic potentials (EPSPs) to interneurons and motor neurons, and indirect evidence suggests that they also transmit via fast EPSPs [39]. Slow EPSPs in IPANs are mimicked by the NK3 receptor agonist senktide [61] and partially blocked by the NK3 receptor antagonist SR142801 [41]. ACh, acting via muscarinic receptors, also elicits slow depolarizing responses in myenteric IPANs [62]. However, a major component of the transmission by IPANs appears to occur via cholinergic fast EPSPs because the application of an antagonist of nicotinic ACh receptors, hexamethonium, at the site of the primary afferent to interneuron synapses reduces ascending reflexes in response to mucosal distortion [63]. Moreover, 5HT_4_ receptor activation facilitates ACh release in the myenteric plexus [64] and may initiate enteric reflexes by acting on the ending IPANs [65]. Therefore, the inhibitory effects of atropine [29], GR113808, adenosine, and SR142801 on ghrelin-induced gastric contractions suggest the importance of IPANs in the regulation of ghrelin activity in motility response. However, we cannot make a firm conclusion because the pharmacological properties of IPANs have been elucidated only for the intestines and not for the stomach thus far. Therefore, a future line of investigation will be to confirm the morphological presence of IPANs in the *S. murinus* stomach with co-localization of receptors for ghrelin, ACh, NK_3_, and 5HT_4_.

**Figure 6:**
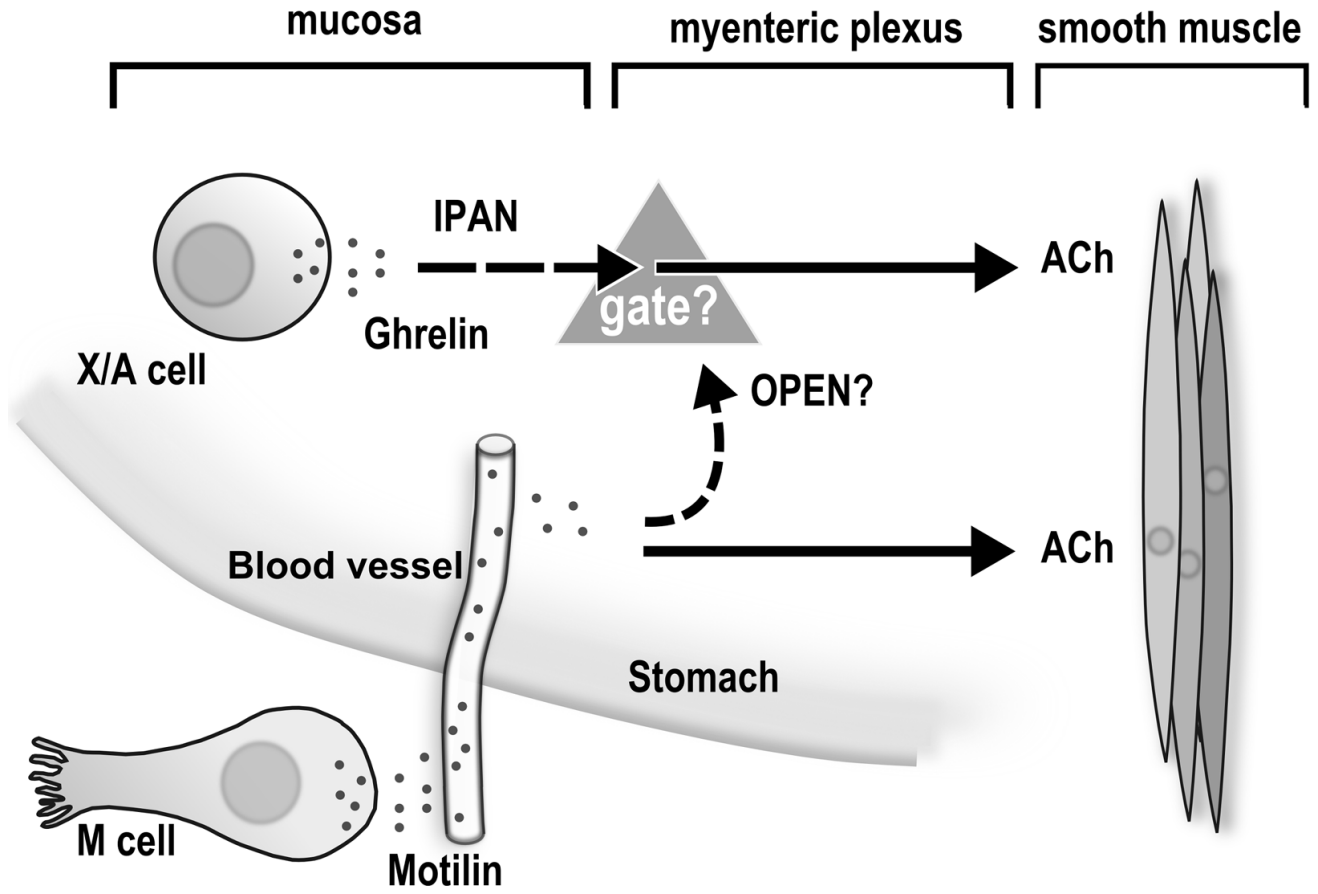
Predicted mechanism of the ghrelin-stimulatory pathway in *Suncus murinus* stomach. Considered with the previous results that motilin (10^−9^ to 10^−7^ M) induces gastric contractions by activating the myenteric plexus [28], the present findings suggest that a very low dose of motilin (10^−10^ M) may initiate an “open gate” effect and restore ghrelin activity via the myenteric plexus. Consequently, ghrelin may transfer signals from the mucosa to the myenteric plexus through involvement of intrinsic primary afferent neurons (IPANs) and regulate gastric contractions through muscarinic cholinergic receptors.

Finally, we concluded that the effect of ghrelin-induced gastric contractions is mediated through postganglionic and preganglionic cholinergic receptors, 5-HT_3_ receptors, α and α_1_ receptors, and opioid receptors; NO synthesis is also involved, and the final mediators of these ghrelin-induced gastric contractions are myenteric cholinergic neurons [28], [29]. Moreover, the mucosa plays an important role in the neural pathway of ghrelin-induced gastric contraction. The reason why pretreatment with a low dose of motilin (10^−10^ M) is needed to induce ghrelin contractions remains unknown. Our predicted mechanism for ghrelin-induced gastric contractions is demonstrated in Figure 6; motilin stimulates gastric contractions through the myenteric plexus [28], whereas ghrelin may stimulate muscarinic cholinergic receptors by passing signals from the mucosa to the myenteric plexus through the stimulation of 5HT_4_, ACh, and NK_3_ release from IPANs. Administration of low doses of motilin may open a gate so that ghrelin can induce gastric contractions in the *S. murinus* stomach. The confirmation of this mechanism may be an interesting finding in the near future. Moreover, the synergistic effects of motilin and ghrelin observed in the present study are probably species-dependent, and clarifying the potential clinical application of these motilin/ghrelin-derived compounds would be interesting.

## Supporting Information

Figure S1 **Effect of adenosine and SR142801 pretreatment on the motilin-induced contractions.** Both the adenosine (10-8.5 M; A) and SR142801 (10^−7^ M; B) has no effect on the motilin-stimulatory pathway. Each value is mean ± SEM (N = 4). •: Control; ▪: antagonist treatment.
(TIF)

Figure S2 **Effect of GR113808 pretreatment on the motilin-induced contractions.** The GR113808 (10^−7^ M) partially inhibited the motilin-stimulatory pathway. Each value is mean ± SEM (N = 12). •: Control; ▴: antagonist treatment.
(TIF)

Table S1 **Comparison between the effects of different receptor antagonist on the motilin- and ghrelin-induced** ***S. murinus*** **gastric contractions.**
(DOCX)
