# Supplementary figures and images for: Mechanism of Ghrelin-Induced Gastric Contractions in Suncus murinus (House Musk Shrew): Involvement of Intrinsic Primary Afferent Neurons
Source: PLoS One. 2013 Apr 2;8(4):e60365. doi: 10.1371/journal.pone.0060365 (PMC3614873; doi:10.1371/journal.pone.0060365)

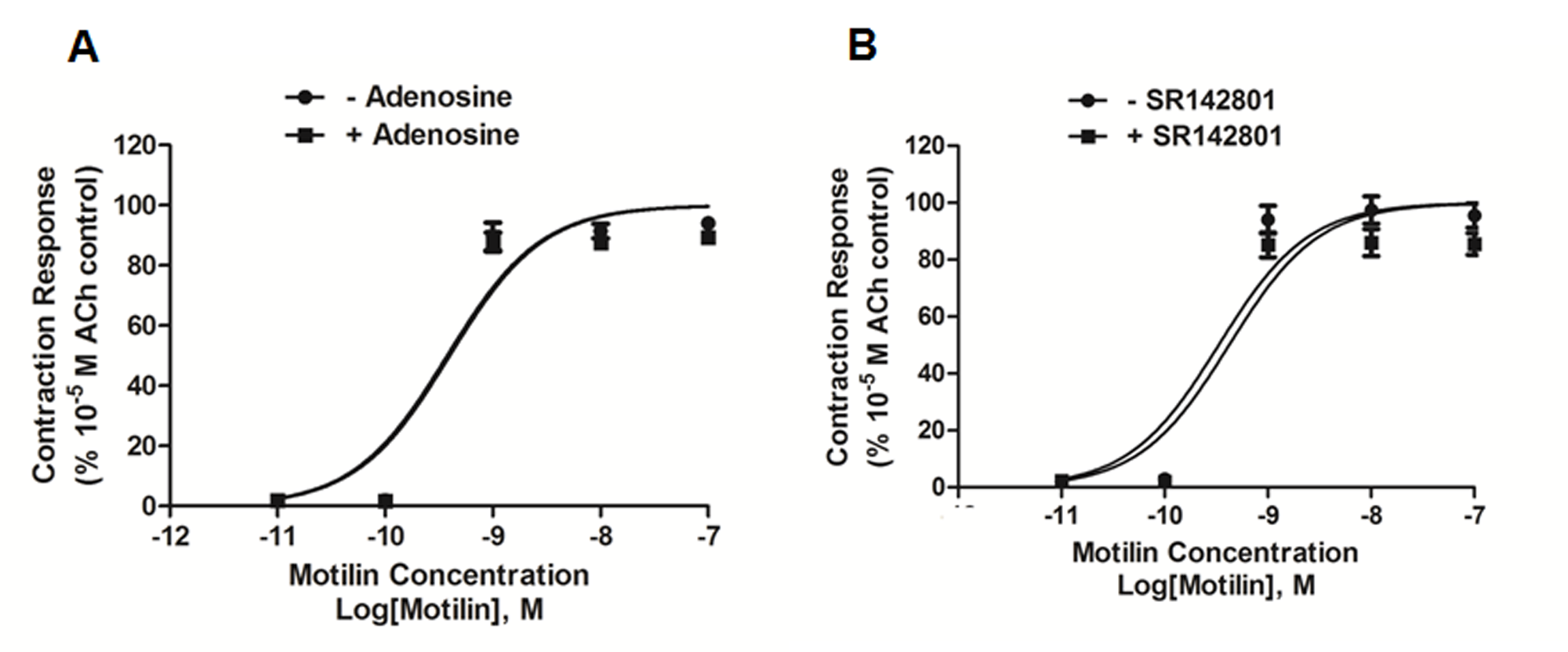

Supplement: Figure S1 — Effect of adenosine and SR142801 pretreatment on the motilin-induced contractions. Both the adenosine (10-8.5 M; A) and SR142801 (10−7 M; B) has no effect on the motilin-stimulatory pathway. Each value is mean ± SEM (N = 4). •: Control; ▪: antagonist treatment. (TIF) [file pone.0060365.s001.tif]

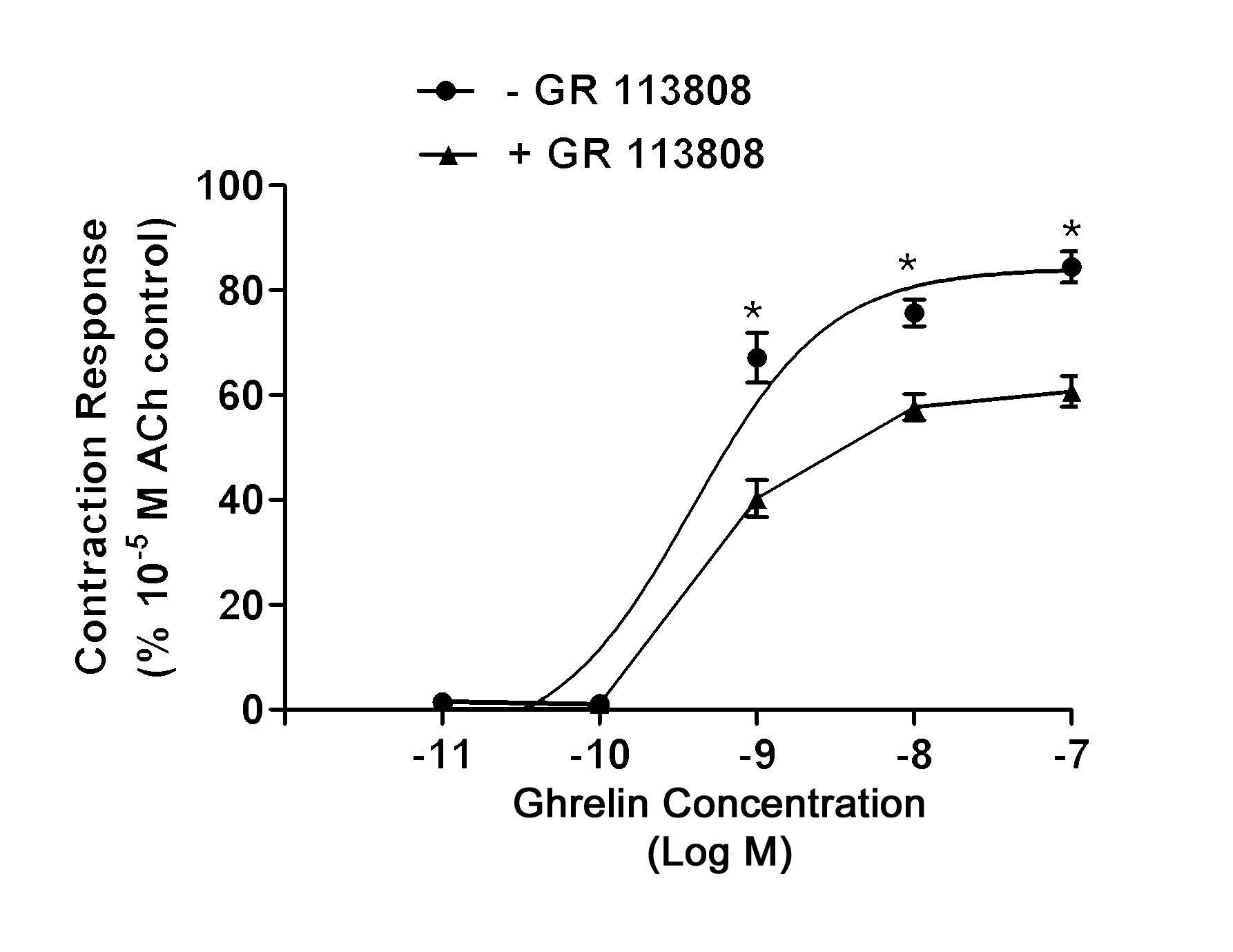

Supplement: Figure S2 — Effect of GR113808 pretreatment on the motilin-induced contractions. The GR113808 (10−7 M) partially inhibited the motilin-stimulatory pathway. Each value is mean ± SEM (N = 12). •: Control; ▴: antagonist treatment. (TIF) [file pone.0060365.s002.tif]
