# Supplementary material for: Mechanism of Ghrelin-Induced Gastric Contractions in Suncus murinus (House Musk Shrew): Involvement of Intrinsic Primary Afferent Neurons
Source: PLoS One. 2013 Apr 2;8(4):e60365. doi: 10.1371/journal.pone.0060365 (PMC3614873; doi:10.1371/journal.pone.0060365)
Supplement: Table S1 — Comparison between the effects of different receptor antagonist on the motilin- and ghrelin-induced S. murinus gastric contractions. (DOCX) [file pone.0060365.s003.docx]

**Table S1:** Comparison between the effects of different receptor antagonist on the motilin- and ghrelin-induced *S. murinus* gastric contractions.

| Antagonists | % of Maximum (mean ±SEM) | | P- value |
| --- | --- | --- | --- |
|  | Effect on the motilin-induced gastric contractions^†^ | Effect on the ghrelin-induced gastric contractions |  |
| Hexamethonium | 42.9 ± 1.5 | 10.1 ± 2.7 | ** |
| Phentolamine | 35.8 ± 2.9 | 18.6 ± 4.8 | * |
| Prazosin | 78.7 ± 3.6 | 22.5 ± 3.9 | ** |
| Ondansetron | 79.4 ± 6.9 | 29.1 ± 3.1 | ** |
| Naloxone | 77.7 ± 1.9 | 22.1 ± 4.1 | ** |

^†^ Data adopted from previous study on motilin-induced gastric contraction [[1](#_ENREF_1)]

* P < 0.05; ** < 0.01

**Reference**

1. Mondal A, Kawamoto Y, Yanaka T, Tsutsui C, Sakata I, et al. (2011) Myenteric neural network activated by motilin in the stomach of Suncus murinus (house musk shrew). Neurogastroenterol Motil 23: 1123-1131.
